# Clinical and computational exploration of red date fruit vinegar: synergistic effects on cardiovascular and type 2 diabetes pathways

**DOI:** 10.3389/fnut.2025.1557733

**Published:** 2025-07-14

**Authors:** Zeshan Ali, Hamad Rafique, Rana Adnan Tahir, Tania Saeed, Muhamad Amir Rasheed, Ishrat Khan, Tawfiq Alsulami, Muqadas Shahzadi, Mariam Laraib Amir, Suleiman A. Althawab, Fangchao Cui

**Affiliations:** ^1^College of Food Science and Technology, Bohai University, Liaoning, China; ^2^National & Local Joint Engineering Research Center of Storage, Processing and Safety Control Technology for Fresh Agricultural and Aquatic Products, Jinzhou, Liaoning, China; ^3^College of Food Engineering and Nutritional Science, Shaanxi Normal University, Xi’an, Shaanxi, China; ^4^Department of Biology, College of Science, Sultan Qaboos University, Muscat, Oman; ^5^Department of Biosciences, COMSATS University Islamabad, Sahiwal Campus, Punjab, Pakistan; ^6^Business School, Division of Management and Administrative Science, University of Education, Lahore, Pakistan; ^7^School of Life Sciences and Biotechnology, Shanghai Jiao Tong University, Shanghai, China; ^8^Department of Food Science and Nutrition, College of Food and Agricultural Sciences, King Saud University, Riyadh, Saudi Arabia; ^9^Department of Zoology, Faculty of Life Sciences, University of Okara, Okara, Punjab, Pakistan; ^10^Department of Bicinformatics and Biotechnology, Government College University Faisalabad, Faisalabad, Pakistan

**Keywords:** date fruit vinegar, phytochemicals, cardiovascular disease (CVD), type 2 diabetes mellitus (T2DM), computational analysis, pulse vacuum drying (PVD)

## Abstract

**Introduction:**

Dates (Phoenix dactylifera), often questioned for their high sugar content, may provide anti-diabetic benefits through their phytochemicals. Date vinegar offers a potentially effective alternative with reduced sugar content and enhanced bioactivity. This study evaluated the effects of date vinegar on glycemic control and lipid profiles in adults with type 2 diabetes mellitus (T2DM) and dyslipidemia while exploring the molecular mechanisms of its bioactive compounds in managing cardiovascular diseases (CVDs) and T2DM.

**Methods:**

A 10-week randomized controlled trial assessed the clinical effects of date vinegar. Complementary experiments explored therapeutic mechanisms through computational analysis and assessed sugar reduction and bioactive preservation under varying drying conditions. Fifty adults with T2DM and dyslipidemia were randomized into two groups: 25 participants received 20 mL of date vinegar daily, while 25 received a placebo. Clinical parameters were measured, including HbA1c, LDL cholesterol, and fasting blood sugar. Computational docking and molecular dynamics simulations investigated interactions of bioactive compounds with key protein targets.

**Results:**

Significant improvements were observed: HbA1c reduced from 6.85 to 6.08%, LDL cholesterol from 121.05 to 111.09 mg/dL, and fasting blood sugar from 168.4 to 147.6 mg/dL (*p* < 0.05). Key compounds with stable protein-ligand complexes confirmed were bound to targets such as ACE, β1AR, hATRs, AR, DPP-IV, and SGLT1. Higher drying temperatures reduced sugar content to match fresh dates but compromised bioactive integrity.

**Conclusion:**

Date vinegar offers a dual target therapeutic strategy for managing T2DM and CVDs, supported by clinical and computational findings.

## 1 Introduction

Cardiovascular diseases (CVDs), driven by atherosclerosis, hypertension, and inflammation, caused 17.8 million deaths in 2017 and are projected to exceed 23 million by 2030, mainly in low- and middle-income countries (1). Protein pathways critical to CVD progression include the renin-angiotensin system (RAS), 3-hydroxy-3-methylglutaryl-coenzyme A reductase (HMGCR), p38 mitogen-activated protein kinase (p38 MAPK), G-protein-coupled receptors (GPCRs, e.g., β1 adrenergic receptor [β1AR], β2 adrenergic receptor [β2AR]), and C-reactive protein (CRP). The RAS influences cardiovascular and renal health, with angiotensin-converting enzyme (ACE) inhibitors and angiotensin II receptor blockers (ARBs) reducing hypertension and inflammation. The angiotensin II type 1 receptor (AT1R) promotes vasoconstriction and cardiac hypertrophy, while the angiotensin II type 2 receptor (AT2R) supports vasodilation. HMGCR inhibition lowers cholesterol, thereby reducing coronary risks. p38 MAPK mediates myocardial apoptosis. GPCR signaling involving β-adrenergic receptor kinase (βARK) worsens heart failure, and CRP predicts cardiovascular events. Cyclooxygenase-2 (COX-2) inhibitors pose risks for CVD (2).

Diabetes mellitus (DM) is a group of metabolic disorders characterized by hyperglycemia and chronic dysfunction of organs, proteins, and enzymes. Addressing its complexity requires multifaceted approaches. Type 2 diabetes mellitus (T2DM), driven by genetic predisposition and factors like obesity, aging, lifestyle, and hormonal imbalances, involves multiple therapeutic targets. Key receptors for T2DM treatment include the insulin receptor (IR), sodium-glucose cotransporter 1 (SGLT1), dipeptidyl peptidase-IV (DPP-IV), protein tyrosine phosphatase 1β (PTP1β), and aldose reductase (AR) (3). IR regulates glucose uptake, with its dysfunction causing insulin resistance in diabetes. SGLT1 facilitates intestinal glucose absorption, inhibiting postprandial glucose. DPP-IV degrades incretins, which modulate insulin and glucagon; DPP-IV inhibitors enhance incretin activity. PTP1β impairs insulin signaling; its inhibition improves sensitivity. AR converts glucose to sorbitol, driving oxidative stress, while AR inhibitors mitigate complications. These pathways provide diverse strategies for T2DM (4). Computational (*in silico*) approaches now enable the identification of drug targets for cardiovascular and metabolic disorders, accelerating drug development by analyzing protein interactions and signaling pathways to design targeted therapies.

Dates are renowned for their flavor and health benefits, containing bioactive compounds such as polyphenols, flavonoids, vitamins, and dietary fiber. Their therapeutic potential, traditionally recognized, is now being extended through the bioavailability-enhancing effects of date vinegar. By fermenting dates into vinegar, these bioactive compounds become more readily absorbed by the body, enhancing their therapeutic properties. This makes date vinegar an even more effective alternative for managing diseases like cancer, heart disease, diabetes, and gastrointestinal disorders. As synthetic drugs for conditions like CVD and T2DM often have side effects, plant-based medicinal systems, including those using date vinegar, are gaining popularity for their affordability, effectiveness, and minimal side effects (5, 6). Understanding the sugar dynamics in dates is crucial for assessing their suitability as a functional food for people with diabetes. Conventional drying methods, like sun or hot air drying, often degrade bioactive compounds and retain higher sugar levels, while modern techniques, like pressure vacuum drying (PVD), preserve bioactive compounds and reduce sugar content, making them ideal for diabetic-friendly products (7).

This study delves into the therapeutic potential of date fruit-derived phytochemicals and date vinegar supplementation in combating CVD and T2DM. Given the overlapping molecular pathways and common target proteins between these two conditions, a promising opportunity exists to develop dual-target therapies that simultaneously address CVD and T2DM. By integrating clinical findings with cutting-edge computational analyses, this research presents a pioneering, holistic approach to unlocking the health benefits of date fruit. The results pave the way for future investigations into date vinegar as a dual-action intervention, with a strong call for more long-term, expansive studies to validate these early findings. While fresh dates were used for vinegar production, an independent drying experiment employing PVD was also conducted to assess the dynamic shifts in sugar content under various temperature conditions. This experiment aims to provide a deeper understanding of the role drying plays in sugar reduction and further evaluates the potential of dates, in their functional forms like vinegar, as a promising solution for managing T2DM.

## 2 Materials and methods

### 2.1 Red date vinegar preparation

Red date vinegar was prepared by steaming a mixture of 1 part sorghum, 1 part bran, and 3 parts water for 2 h. Fresh red dates, the main component, were added at a ratio of 1 part date to 3 parts of the combined sorghum-bran mixture. Alcoholic fermentation was initiated by inoculating the mixture with *Saccharomyces cerevisiae* and allowing it to ferment for 7–10 days at 25–30°C, during which the yeast converted sugars into ethanol. This was followed by acetic acid fermentation through the inoculation of *Acetobacter* species, which oxidized the ethanol to acetic acid over 10–14 days at 30–35°C. To enhance flavor and inhibit microbial contamination, the mixture was smoked for 9 days. It was then boiled, sieved, sun-dried, and aged naturally for 6 months, during which seasonal summer sunlight further developed its flavor profile.

### 2.2 Study design and participant characteristics

This 10-week, randomized, placebo-controlled clinical trial was conducted to evaluate the effects of date vinegar on glycemic control and lipid profiles in patients with T2DM and hyperlipidemia. A total of 50 eligible participants (20 females and 30 males), aged 30–60 years and weighing 50–70 kg, were recruited from Al-Rashid Clinic, Lahore, Pakistan. All participants had fasting blood sugar (FBS) levels above 120 mg/dL and low-density lipoprotein (LDL) cholesterol levels between 200 and 240 mg/dL at baseline.

Participants were randomly assigned to one of two groups (*n* = 25 each). The intervention group received 20 mL of date vinegar daily, consumed with breakfast or bedtime. The control group received a placebo of 5 mL of honey, 15 mL of water, and lemon juice to match vinegar acidity, but without bioactive compounds. The vinegar and placebo were pre-measured and packed in 20 mL sterile, food-grade plastic vials with tight-sealing caps to ensure hygienic and accurate daily consumption.

All participants were instructed to continue their usual diet and medication regimens for diabetes and hyperlipidemia throughout the study. To reduce confounding, stratified randomization was applied based on medication type. Baseline demographic and clinical data are presented in Table 1, and daily nutrient intake at baseline and post-intervention is provided in Table 2. No participants dropped out or reported serious adverse effects during the study.

**TABLE 1:** Patient characteristics in the dates vinegar and placebo group.

Parameter	Group 1	Group 2	*p*-value
Age (years)	46.4 ± 1.5	47.2 ± 1.8	0.094
Female/Male (n)	11/14	9/16	0.27
BMI (Kg/m^2^)	24.3 ± 0.5	23.7 ± 0.7	0.001
Systolic BP (mmHg)	121.8 ± 1.4	121.3 ± 1.8	0.279
Diastolic BP (mmHg)	75.5 ± 1.3	76.2 ± 1.5	0.084
Cholesterol (mg/dL)	225.9 ± 3.4	219.1 ± 3.0	0.031
Triglycerides (mg/dL)	200.5 ± 4.3	197.5 ± 3.7	0.011
HDL-C (mg/dL)	43.2 ± 3.9	42.1 ± 4.8	0.378
LDL-C (mg/dL)	121.5 ± 4.9	118.1 ± 4.6	0.015

Baseline characteristics of participants in group 1 and group 2. Values are presented as mean ± standard deviation (SD). *p*-values were calculated using an independent two-sample *t*-test assuming unequal variances. A *p*-value < 0.05 was considered statistically significant. Group sizes: Group 1 (*n* = 25; 11 females, 14 males), Group 2 (*n* = 25; 9 females, 16 males).

**TABLE 2:** Mean daily intake of selected nutrients at baseline and after intervention.

Nutrients	Vinegar group (before)	Vinegar group (after)	Placebo group (before)	Placebo group (after)	*p*-value (Vinegar group)	*p*-value (Placebo group)
Energy (kcal)	1543 ± 41	1462 ± 32	1462 ± 58	1498 ± 33	0.137	0.135
Carbohydrate (g)	253.6 ± 6.9	250.3 ± 5.9	248.1 ± 6.6	249.2 ± 5.3	0.462	0.387
Protein (g)	61.5 ± 4.5	60.1 ± 4.0	57.9 ± 2.6	55.7 ± 2.2	0.268	0.164
Total fat (g)	39.1 ± 4.8	37.5 ± 4.5	37.0 ± 2.7	36.2 ± 2.9	0.110	0.198
Dietary fiber (g)	23.3 ± 1.2	21.2 ± 1.5	19.5 ± 1.5	19.7 ± 1.7	0.002	0.497
Saturated fat (g)	4.7 ± 0.7	4.2 ± 0.4	3.8 ± 0.4	4.0 ± 0.6	0.405	0.232
Monounsaturated fat (g)	7.3 ± 0.9	7.0 ± 0.3	6.6 ± 0.6	6.5 ± 0.8	0.398	0.416
Polyunsaturated fat (g)	4.1 ± 0.7	3.6 ± 0.9	3.4 ± 0.7	3.8 ± 0.5	0.524	0.312
Cholesterol (mg)	204.5 ± 6.9	189.9 ± 6.8	216.3 ± 5.6	220.2 ± 6.0	0.002	0.220

Values are presented as mean ± standard deviation (SD). “Before” refers to baseline measurements taken prior to the intervention, while “After” refers to measurements recorded at the end of the 10-week intervention period. *p*-values represent the significance of differences within each group (vinegar or placebo) by comparing pre- and post-intervention values. A *p*-value < 0.05 was considered statistically significant.

### 2.3 Inclusion and exclusion criteria

Participants were included if they met the following criteria: diagnosed with type 2 diabetes mellitus and hyperlipidemia, aged between 30 and 60 years, fasting blood sugar > 120 mg/dL, LDL cholesterol between 200 and 240 mg/dL, body weight between 50 and 70 kg, and willingness to provide written informed consent.

Exclusion criteria included any history of hepatic, renal, cardiovascular, or asthmatic diseases; known vinegar intolerance; alcohol consumption; or participation in another clinical trial during the same period.

### 2.4 Ethical considerations and participant consent

All participants were fully informed of the study’s objectives, methodology, potential risks, and benefits prior to inclusion. Written informed consent was obtained from each participant, who was also assured of data confidentiality and the right to withdraw from the study at any stage without penalty. The study protocol was approved by the Institutional Review Board of Minhaj University Lahore, Pakistan (Approval No: MUL/F&N/Ref-560), and all procedures were conducted following the Declaration of Helsinki.

### 2.5 Blood collection and biochemical analysis

Selected patients underwent a thorough clinical evaluation after providing written informed consent. Blood samples (5 mL each) were collected at baseline, 5 weeks, and 10 weeks. After allowing the samples to clot, they were centrifuged at 3,500 rpm for 15 min, and serum was obtained. Biochemical and hematological parameters were analyzed using an automated biochemistry analyzer (Metro Lab 2300 PLUS, Vital Scientific B.V., Netherlands). Fasting blood sugar (FBS), triglycerides (TG), and high-density lipoprotein (HDL) concentrations were measured according to the manufacturer’s instructions. Low-density lipoprotein (LDL) concentration was calculated using the Friedewald formula:


(1) L⁢D⁢L-C=T⁢C-H⁢D⁢L-C-T⁢G/5

### 2.6. Computational analysis

#### 2.6.1. Ligand preparation

An extensive literature review was conducted to retrieve the wide variety of phytochemicals derived from date fruit and its vinegar with potent activity. The 2D chemical structures of all phytochemicals were drawn and subsequently converted into 3D coordinates by employing ChemDraw Office (8). The phytochemicals were subjected to energy minimization using the UCSF Chimera 1.17.3 (9) and 3d structures in .pdb format for docking analysis. The Auto Dock Tool assessed all compounds’ Gasteiger charges and rotatable bonds (10).

#### 2.6.2 Protein preparation

The potential targets of CVD and T2DM were retrieved through an extensive literature review, and five promising targets were shortlisted for both CVD and T2DM based on their significant association with these disorders (Table 3).

**TABLE 3:** Selected targeted receptor proteins associated with CVD and T2DM.

Disease	Pathological conditions	Target proteins	PDB ID	References
CVD	Atherosclerosis and coronary artery disease	Angiotensin-converting enzyme	1O8A	(11, 12)
	Atherosclerosis and coronary artery diseases	Human Angiotensin receptor	4YAY	(12, 13)
	Myocardial infarction	P38 Mitogen-activated protein kinase	4DLI	(12, 14)
	Coronary artery disease	HMG-Co A reductase	1HW9	(12, 15)
	Chronic heart failure	ß-1-Adrenergic receptor	2YCW	(12, 16)
T2DM	Regulation of glucose homeostasis	Insulin Receptor	1IR3	(17, 18)
	Reabsorption of glucose	Sodium-glucose cotransporter 1	3DH4	(12, 19)
	T2DM	human dipeptidyl peptidase-IV	3F8S	(12, 18)
	T2DM	protein tyrosine phosphatase 1β	2F70	(18, 20)
	T2DM	Human Aldose Reductase	3S3G	(21)

The crystal structures of target receptor proteins of CVD, such as ACE (PDB ID # 1O8A), hATR (PDB ID # 4YAY), MAPK p38 (PDB ID # 4DLI), HMGCR (PDB ID # 1HW9), and β1AR (PDB ID # 2YCW) were accessed from protein databank (PDB) (22). The crystal structures of T2DM target proteins, including IR (PDB ID # 1IR3), SGLT1 (PDB ID # 3DH4), DPP-IV (PDB ID # 3F8S), PTP1β (PDB ID # 2F70), and AR (PDB ID # 3S3G) were also retrieved from PDB (22). The crystal structures of target proteins were prepared by removing all non-standard residues and extra polypeptide chains through UCSF Chimera 1.17.3 (9). Structure preparation also involved the addition of polar hydrogens and charges before molecular docking analysis.

#### 2.6.3 Molecular docking analyses

Molecular docking analyses of date fruit and its vinegar-derived phytochemicals with CVD and T2DM target proteins were performed using AutoDock Vina (10). Docking predicts the binding affinity between receptors and ligands by evaluating potential conformations and orientations through scoring functions. MGL Tools converted 3D structures of receptors and ligands into pdbqt files, including atomic coordinates, charges, and atom types (23). The grid box defined receptor protein coverage, ensuring all active regions were targeted. AutoDock Vina, with an exhaustiveness of eight and twenty output solutions, determined the binding potential for CVD and T2DM proteins. The top solutions with the lowest binding energy were selected for visualization and interaction analysis using UCSF Chimera 1.17.3 and MoE (9, 24).

#### 2.6.4 Molecular dynamics simulation

Ligand-protein complexes with favorable binding affinities were analyzed through 20 ns molecular dynamics (MD) simulations using Desmond-Maestro (25, 26). The top two complexes underwent an additional 100 ns simulation for structural insights and stability. Protein-ligand complexes were preprocessed using Maestro’s optimization and minimization tools. Systems were prepared with the System Builder tool, immersed in TIP3P water models, and simulated with the OPLS 2005 force field. Physiological conditions were mimicked by adding 0.15 M NaCl, and MD simulations were conducted under stable thermodynamic conditions (300 K, 1 atm) (27). Stability was assessed by root mean square deviation (RMSD), and further analyses, including RMSF, ligand conformational changes, and intermolecular interactions, were performed. Binding free energies (ΔG) were calculated using MMGBSA after the 100 ns simulation.

#### 2.6.5 *In Silico* pharmacokinetic and drug-likeness analyses

The physicochemical properties of phytochemicals were predicted using ADMETlab 2.0 (28) to assess their absorption, bioavailability, distribution, and drug-likeness features. The primary method to predict oral bioavailability, permeability, and absorption is Lipinski’s “Rule of Five” (RO5). Compounds should have molecular weight ≤ 500, hydrogen bond acceptors ≤ 10, hydrogen bond donors ≤ 5, and a logP value ≤ 5 for better absorption. RO5 was applied to these phytochemicals to determine RO5 violations and conduct subsequent analyses.

### 2.7. PVD and total sugar content measurement

Red dates were dried using PVD at 65, 70, and 75°C with a pulse ratio of 15:4. This method combines vacuum pressure and heat to minimize drying time while preserving the quality of the dates. The anthrone-sulfuric acid method measured the total sugar content in red date slices. A 2.0 g sample was extracted with 80% ethanol, centrifuged, and the supernatants combined and diluted. A standard glucose curve (y = 0.4866x + 0.0007, R^2^ = 0.9995) was generated, and the total sugar content was determined by measuring absorbance at 620 nm after treating the sample with anthrone reagent. The sugar concentration was calculated using the formula:


(2) $$W=\frac{C\times n\times V_{1}}{V_{2}\times m\times 1000}$$

### 2.8. Statistical analysis

Data analysis was performed using SPSS (version 21.0). One-way ANOVA was used to compare group differences with Duncan’s test for *post hoc* analysis. Paired *t*-tests assessed between-group variations. Statistical significance was set at *p* < 0.05, and results are presented as mean ± standard deviation (SD).

## 3 Results and discussion

Earlier studies have primarily focused on the acetic acid content of vinegar, with less emphasis on other phytochemicals present. Our previous studies (5, 6, 29) have shown that commercially available date vinegar and date vinegar-based beverages improve lipid profiles and diabetes management. However, the *in vivo* mechanisms and suitability of date fruit and vinegar for diabetic hyperlipidemic patients remain unclear. This study aimed to evaluate the effects of date vinegar on diabetic dyslipidemic adults, focusing on enhancing its bioavailability. By assessing its impact on key diabetic and lipid markers, we explored its *in vivo* mechanisms and potential protein targets using *in silico* approaches, providing a clearer understanding of its therapeutic potential in managing diabetes and metabolic disorders.

### 3.1 Effects of date vinegar on glycemic control and lipid profile

Table 4 summarizes diabetic and lipid markers, highlighting significant improvements in the vinegar group except for non-significant triglycerides and VLDL changes.

**TABLE 4:** Changes in lipid and diabetic markers.

Parameter	Time point	Vinegar group (Mean ± SD)	Placebo group (Mean ± SD)	*p*-value (Vinegar Group)
Cholesterol (mg/dL)	Baseline	225.09 ± 3.40	219.10 ± 3.00	p < 0.05
	Week 5	218.90 ± 3.10	217.30 ± 2.80	
	Week 10	213.14 ± 3.00	218.40 ± 3.20	
LDL (mg/dL)	Baseline	121.05 ± 4.90	118.10 ± 4.60	p < 0.01
	Week 5	115.09 ± 3.80	117.60 ± 4.50	
	Week 10	111.09 ± 3.20	119.90 ± 4.30	
Triglycerides (mg/dL)	Baseline	200.05 ± 4.30	197.50 ± 3.70	NS
	Week 5	198.21 ± 3.50	198.40 ± 3.10	
	Week 10	196.17 ± 3.40	198.90 ± 3.20	
VLDL (mg/dL)	Baseline	46.90 ± 4.70	43.64 ± 4.20	NS
	Week 5	45.10 ± 3.80	43.62 ± 3.90	
	Week 10	44.44 ± 3.10	44.48 ± 3.50	
HDL (mg/dL)	Baseline	43.20 ± 3.90	42.10 ± 4.80	p < 0.05
	Week 5	44.10 ± 2.90	42.70 ± 2.60	
	Week 10	46.50 ± 3.30	41.40 ± 2.90	
HbA1c (%)	Baseline	6.85 ± 0.30	6.50 ± 0.40	p < 0.05
	Week 5	6.70 ± 0.20	6.60 ± 0.30	
	Week 10	6.08 ± 0.20	6.65 ± 0.30	
FBS (mg/dL)	Baseline	168.40 ± 6.40	162.50 ± 5.60	p < 0.01
	Week 5	157.60 ± 5.61	171.50 ± 5.73	
	Week 10	147.60 ± 4.80	174.40 ± 4.23	

Values are presented as mean ± SD at baseline, week 5, and week 10 for both vinegar and placebo groups. P-values reflect statistical significance between baseline and week 10 within the vinegar group. NS = not significant.

The results for HbA1c showed a reduction in the dates vinegar group, which decreased from 6.85 ± 0.3% to 6.08 ± 0.2% (*p* < 0.05), whereas the placebo group exhibited only a slight increase from 6.50 ± 0.4% to 6.65 ± 0.3%. This reduction in HbA1c indicates that red date vinegar may improve long-term blood glucose control in individuals with T2DM. Regarding FBS, the vinegar group exhibited a significant decrease from 168.4 ± 6.4 mg/dL to 147.6 ± 4.8 mg/dL (*p* < 0.01), while the placebo group showed an increase from 162.5 ± 5.6 mg/dL to 174.4 ± 4.23 mg/dL. This decrease in HbA1c and FBS is consistent with the findings that showed Hayani date peel powder and its methanolic extract significantly reduced glycemia and lipidemia in streptozotocin-induced diabetic rats. Serum glucose decreased by 70.48% with the extract and 53.37% with the powder. HbA1c levels dropped to 5.69% ± 0.43 and 5.77% ± 0.48, respectively, compared to 9.32% ± 0.98 in untreated diabetic rats, demonstrating their potential in managing hyperglycemia and lipid metabolism (30). While one study (100 T2DM subjects: 39 male and 61 female) found that consuming a low dose of three dates daily for 16 weeks had minimal impact on glycemic control, with no significant change in HbA1c (Δ = 0.087%, *p* > 0.05), it did lead to a substantial reduction in total cholesterol (Δ = −0.209 mmol/L, *p* < 0.05) and LDL (Δ = −0.171 mmol/L). This suggests that while dates improve lipid profiles, their low glycemic index limits their effect on glucose levels (31).

The vinegar group also significantly reduced total cholesterol from 225.09 ± 3.4 mg/dL to 213.14 ± 3.0 mg/dL (*p* < 0.05). In contrast, the placebo group showed minimal change from 219.1 ± 3.0 mg/dL to 218.4 ± 3.2 mg/dL. In terms of LDL, a significant decrease was observed in the vinegar group from 121.05 ± 4.9 mg/dL to 111.09 ± 3.2 mg/dL (*p* < 0.01), while the placebo group exhibited only minor changes from 118.1 ± 4.6 mg/dL to 119.9 ± 4.3 mg/dL. Elevated LDL is a significant risk factor for cardiovascular disease, and the reduction observed in our study suggests that red date vinegar may be beneficial for reducing cardiovascular risk. Our results align with a study that assessed the effects of consuming 5–7 Zahdi dates daily for 21 days in 24 control subjects (12 male, 12 female, mean age 42.0 ± 8.69 years) and 20 type 2 diabetes patients (10 male, 10 female, HbA1c 9.25 ± 2.02%, age 47.7 ± 9.33 years). The study found a significant decrease in total cholesterol (from 6.94 ± 0.84 mmol/L to 5.32 ± 0.41 mmol/L) and a substantial increase in glutathione and vitamin E in diabetic patients compared to the control group (32). A recent study showed that red date vinegar reduced body weight by 19.92%, serum TC, TG, and LDL-C by 25.09, 26.83, and 11.66%, respectively, while increasing HDL-C by 1.44 times. It also decreased AST and ALT by 26.36 and 34.87% and improved antioxidant levels (SOD and GSH-Px) by 1.35- and 1.60-fold, respectively. Malondialdehyde levels dropped by 33.21% (33).

In contrast, no significant differences were observed in triglycerides or very low-density lipoprotein (VLDL) levels between the vinegar and placebo groups (*p* > 0.05 for both parameters). In line with our findings, a pilot trial with 10 healthy non-smokers consuming 100 g/day of either Medjool or Hallawi dates for 4 weeks showed no significant effect on triglyceride levels with Medjool dates, while Hallawi dates led to a 15% reduction in triglycerides. However, the Hallawi group had higher baseline triglycerides, and the results were based on a bar graph, limiting precise comparison (34). This suggests that date varieties may have varying impacts on triglycerides, with limited effects for some types. Finally, the vinegar group showed a significant increase in HDL levels from 43.2 ± 3.9 mg/dL to 46.5 ± 3.3 mg/dL (*p* < 0.05), while the placebo group experienced a slight decrease from 42.1 ± 4.8 mg/dL to 41.4 ± 2.9 mg/dL. This increase in HDL aligns with findings from a 6-week randomized clinical trial, where participants consuming 70 g of Ajwa daily showed significant reductions in total cholesterol (–22.00 ± 4.23 mg/dL), triglycerides (–15.57 ± 4.02 mg/dL), and LDL (–11.38 ± 1.79 mg/dL), along with a 3.19 ± 0.80 mg/dL increase in HDL (*p* = 0.0001). The control group did not change their diet or lifestyle (35). The increase in HDL, known for its protective role in cardiovascular health, may contribute to the health benefits observed in the vinegar group.

### 3.2 Molecular interaction and simulation results

This study utilized computational biology approaches, including molecular docking and molecular dynamics simulations, to investigate the interaction of date and its vinegar-derived phytochemicals with key protein targets associated with CVD and T2DM. Molecular docking identified several promising compounds with strong binding potential to critical CVD and T2DM proteins, particularly pectin, yamogenin acetate, diosgenin, zeaxanthin, and antheraxanthin. These findings align with previous research emphasizing the cardiometabolic benefits of bioactive compounds from plant sources. For instance, one study has highlighted the significant role of carotenoids, such as zeaxanthin, in reducing oxidative stress and improving endothelial function, supporting our findings of zeaxanthin’s notable binding with ACE and AR (36).

### 3.3 Molecular docking analysis

An extensive literature review retrieved 108 distinct phytochemicals from date fruit and vinegar, including phenolic acids (21), flavonoids (9), carbohydrates (3), fibers (3), minerals (12), enzymes (1), carotenoids (6), amino acids (8), fatty acids (4), vitamins (6), phytosterols (15), phytoestrogens (11), natural compounds (4), and secondary metabolites (5). Various studies have shown that these diverse compounds possess cardioprotective and anti-diabetic properties, offering a broad therapeutic potential. For example, diosgenin, identified in our research with strong ACE binding, has previously been reported to exert antihypertensive effects by inhibiting ACE activity, a critical mechanism in managing hypertension in CVD (37).

Ligand preparation involved retrieving chemical information from PubChem and drawing 2D chemical structures using ChemDraw Ultra, followed by conversion into 3D structural coordinates and energy minimization to eliminate steric clashes. This process prepared the phytochemicals library for docking analyses against CVD and T2DM targets. Recent studies have employed similar computational methodologies (38), validating their effectiveness in predicting ligand-receptor interactions before experimental testing.

The co-crystallized structures of CVD-related proteins, such as ACE (PDB ID: 1O8A), β1AR (PDB ID: 2YCW), and hATRs (PDB ID: 4YAY), were retrieved and docked with date-derived compounds. These targets are linked to conditions like myocardial infarction and chronic heart failure. Our results revealed that phytochemicals like antheraxanthin and diosgenin strongly bind to β1AR and ACE, consistent with studies highlighting these proteins’ roles in reducing cardiac stress and inflammation (39).

For T2DM, docking analyses of targets such as SGLT1 (PDB ID: 3DH4), DPP-IV (PDB ID: 3F8S), and AR (PDB ID: 3S3G) showed promising interactions with date-derived compounds. Notably, pectin and yamogenin acetate exhibited high binding affinities with DPP-IV and AR, respectively. These results align with findings where natural DPP-IV inhibitors enhanced glycemic control in T2DM patients (40).

Docking analyses using AutoDock Vina identified the top 26 compounds with strong binding potentials, which were then visualized and analyzed for binding interactions (Table 5). Visualization and analysis of these interactions using UCSF Chimera and MoE tools further confirmed their stability. Similar approaches have been reported earlier where computational predictions closely matched *in vivo* results, strengthening our study’s reliability in identifying novel therapeutic candidates (41).

**TABLE 5:** Binding energies of the top-selected phytochemicals from date fruit vinegar to T2DM and CVD-targeted proteins.

Phytochemicals	CVD Targets (Binding energies) (Kcal/mol)					T2DM targets (Binding energies) (Kcal/mol)				
**Ligands**	**HMGCR**	**β1AR**	**MAPK p38**	**hATR**	**ACE**	** IR**	**PTP1β**	**SGLT1**	**DPP-IV**	** AR**
Rutin	−7.8	−9.1	−9.2	−8.8	−9.8	−9.4	7.3	−7.8	−8.7	−8.9
Pectin	−8.5	−9	−8.8	−9.5	−12.2	−9.2	−9	−9.3	−10.7	−9.6
Pelargonin	−8.4	−9.7	−7.5	−9.0	−9.7	−8.9	−7.1	−7.1	−8.7	−8.3
Estrone	−7.6	−8.9	−8.4	−8.7	−9.5	−8.8	−8.8	−8	−8.1	−8.9
Yamogenin_acetate	−8.1	−9.2	−9.5	−8.7	−11.4	−8.7	−7.7	−9	−9	−10.4
Diosgenin	−7.8	−8.8	−10.1	−10.3	−11.2	−8.6	−8.2	−9.6	−9.9	−9.9
Catechin	−6.8	−8.7	−7.9	−8.1	−8.2	−8.5	−7.2	−8.4	−7.6	−9.8
Coumestrol	−7.4	−8.3	−8.3	−9.1	−8.7	−8.5	−7.7	−8.8	−8.7	−8.8
Coumestans	−7.0	−10.0	−10.6	−9.2	−8.5	−8.4	−7.3	−8	−7.9	−9.2
Diosmetin_1	−8.0	−9.4	−8.1	−9.1	−9.8	−8.4	−7.7	−8	−9	−9.4
Diosmetin_2	−7.5	−8.7	−8.2	−8.5	−9.4	−8.4	−8.3	−9.5	−8.7	−9.4
Lutein	−7.1	−8.1	−6.7	−10.2	−10.5	−6.8	−8.2	−8.4	−9.7	−9.1
Zeaxanthin	−7.5	−8.0	−7.9	−10.6	−10.5	−7.9	−8.1	−9.1	−9.3	−10.2
Matairesinol	−7.0	−7.8	−7.3	−8.6	−8.5	−7.5	−7.9	−8.2	−8.3	−8.5
Procyanidin	−7.9	−8.2	−8.0	−9.9	−10.4	−7.9	−7.9	−8	−9.1	−8.4
Daidzein	−6.7	−8.8	−9.8	−7.8	−8.0	−7.5	−7.8	−7.7	−8.1	−8.5
Antheraxanthin	−7.8	−10.9	−9.0	−10.1	−10.5	−7.6	−7.5	−9.9	−9.3	−9.8
Folic acid	−7.4	−9.8	−8.8	−9.2	−9.5	−7.9	−7.5	−9.8	−8.8	−9.4
Lupenone	−7.7	−8.5	−8.0	−10.1	−9.8	−8.4	−7.2	−9.3	−8.6	−9.2
Lupeol	−6.7	−8.3	−7.6	−8.4	−9.5	−7.8	−7.3	−9.1	−8.5	−9.1
Neoxanthin	−6.8	−8.9	−6.4	−8.8	−10.0	−6.3	−6.4	−9.6	−7.9	−10.4
Violaxanthin	−8.2	−9.4	−8.4	−9.0	−10.2	−6.6	−7.3	−9.6	−9	−9.6
Stigmastan−3 _5-diene	−6.6	−7.6	−7.5	−9.6	−8.8	−6.4	−6.6	−9.3	−8.3	−7.8
Procyanidin_beta_(4−8)	−8.3	−8.2	−8	−8.6	−9.8	−7.6	−7.5	−8	−9.1	−9
24-methylene cycloartenol	−7.9	−8.1	−7.8	−10.5	−10.1	−6.9	−6.8	−8.4	−7.8	−10.2
Formononetin	−7.0	−9.6	−7.8	−8.0	−8.7	−7.6	−7.3	−7.9	−8.3	−9.9

Our study’s integration of computational and pathway-based approaches validates the identified compounds and emphasizes their potential *in vivo* applications. This comprehensive strategy surpasses previous studies focused solely on *in vitro* or *in silico* analyses, paving the way for future experimental validation and clinical trials.

**FIGURE 1:**
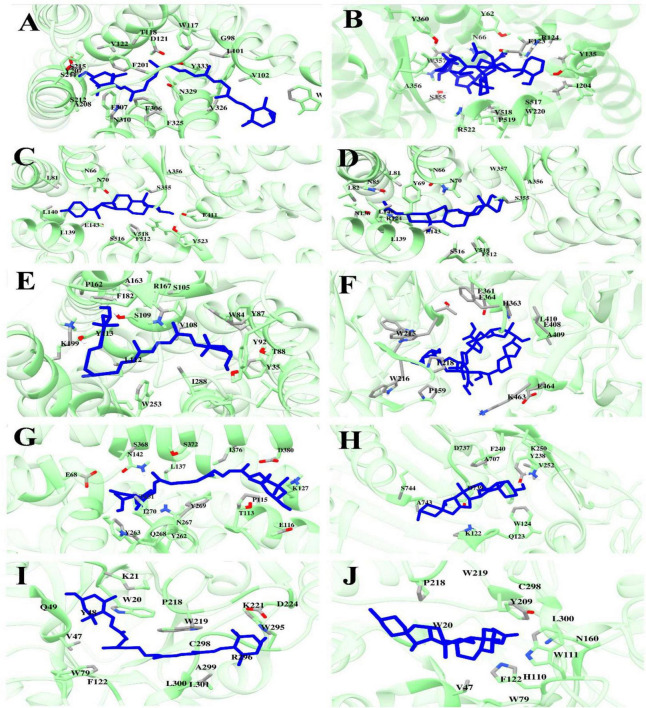
Molecular docking analyses revealed the binding interactions of date vinegar-derived phytochemicals with CVD and T2DM protein targets. Ligands (blue sticks) are shown in the active sites of their respective target proteins (green ribbon structures), with key interacting amino acid residues labeled. **(A)** β1AR–antheraxanthin, **(B)** ACE–pectin, **(C)** ACE–diosgenin, **(D)** ACE–yamogenin acetate, **(E)** hATRs–zeaxanthin, **(F)** DPP-IV–pectin, **(G)** SGLT1–antheraxanthin, **(H)** DPP-IV–diosgenin, **(I)** AR–zeaxanthin, and **(J)** AR–yamogenin acetate. The results highlight the hydrogen bonding, hydrophobic contacts, and favorable positioning within the catalytic pockets, supporting the potential bioactivity of these compounds in modulating cardiometabolic targets.

The binding interactions of selected phytochemicals docked with therapeutic targets of CVD and T2DM are visualized and determined by UCSF Chimera 1.17.3. The Binding interactions of CVD docked complexes with phytochemicals, including β1AR with antheraxanthin, hATRs with zeaxanthin, ACE with diosgenin, ACE with pectin, and ACE-yamogenin acetate, are presented in Figures 1A–E. The binding interactions of T2DM proteins complex with phytochemicals, including SGLT1 with antheraxanthin, human DPP-IV with diosgenin, human DPP-IV with pectin, human AR with yamogenin-acetate, and human AR with zeaxanthin, are presented in Figures 1F–J.

### 3.4 Molecular dynamics simulation analysis

**FIGURE 2:**
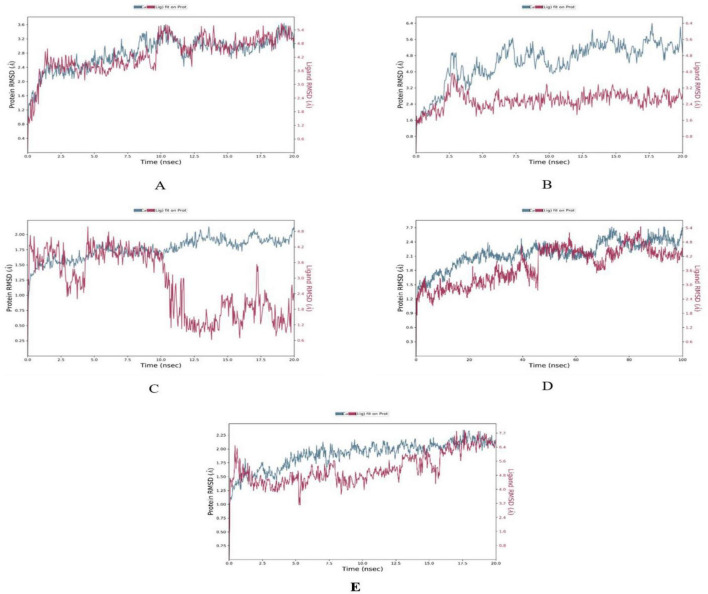
Root Mean Square Deviation (RMSD) plots depicting the structural stability of CVD-related protein–ligand complexes during molecular dynamics (MD) simulations. Each graph displays the RMSD of the protein backbone (blue line) and the ligand (red line) over the simulation time. **(A)** β1AR–antheraxanthin, **(B)** hATRs–zeaxanthin, **(C)** ACE–diosgenin, **(D)** ACE–pectin (100 ns), and **(E)** ACE–yamogenin acetate. The Y-axis represents RMSD values in Å (Ångström), indicating the extent of deviation from the reference structure. Consistently low or plateaued RMSD values indicate stable binding interactions between the phytochemicals and their respective target proteins.

The MD simulations conducted on the selected CVD and T2DM complexes provided detailed insights into the structural dynamics of the protein-ligand interactions. The stability of the backbone structures for most complexes, as indicated by the root mean square deviation (RMSD) values, with deviations falling within an acceptable range (1–3 Å), as reported in similar studies (42). Specifically, the β1AR complex with antheraxanthin demonstrated an RMSD of 2.77 Å, showing good stability. In contrast, the higher RMSD observed for ACE complexes with pectin and yamogenin acetate (3.81 Å and 5.19 Å, respectively) suggested initial instability (Figures 2A–E).

**FIGURE 3:**
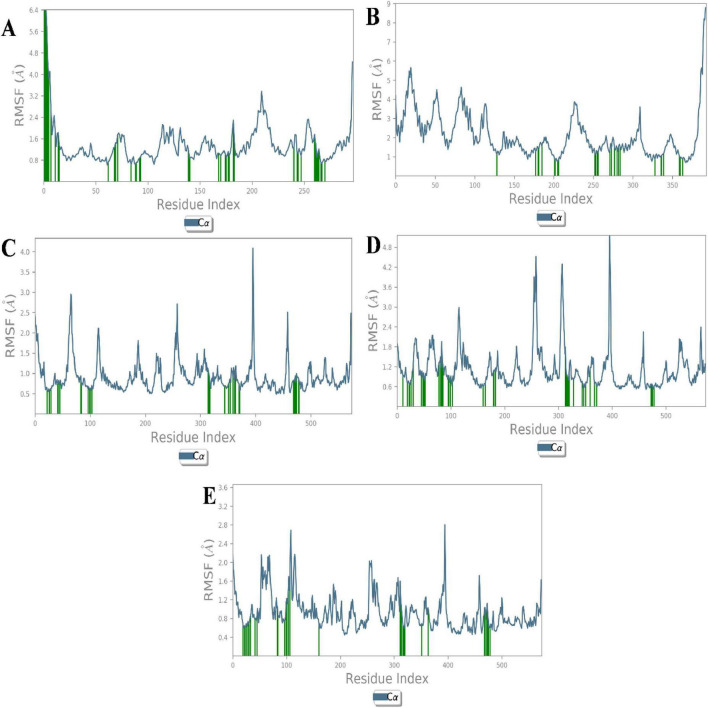
Root Mean Square Fluctuation (RMSF) analysis of CVD-related protein–ligand complexes. The plots represent the flexibility of each residue’s Cα atom throughout the simulation period, indicating atomic displacement around the mean position. **(A)** β1AR–antheraxanthin, **(B)** hATRs–zeaxanthin, **(C)** ACE–diosgenin, **(D)** ACE–pectin, and **(E)** ACE–yamogenin acetate. Peaks in the graph denote flexible loop regions, while troughs correspond to stable secondary structure elements. Consistently low RMSF values near the binding regions suggest stable interactions between the ligands and their respective targets.

Root mean square fluctuation (RMSF) analysis further revealed that fluctuations in specific residues were consistent with findings from similar studies on G-protein-coupled receptors and ACE, where residues in particular regions of the protein backbone exhibited higher flexibility. For instance, fluctuations in the β1AR complex with antheraxanthin were observed in regions such as residues 66–79, which correlates with residues found to be dynamic in other β1AR-ligand simulations (43) (Figures 3A–E).

**FIGURE 4:**
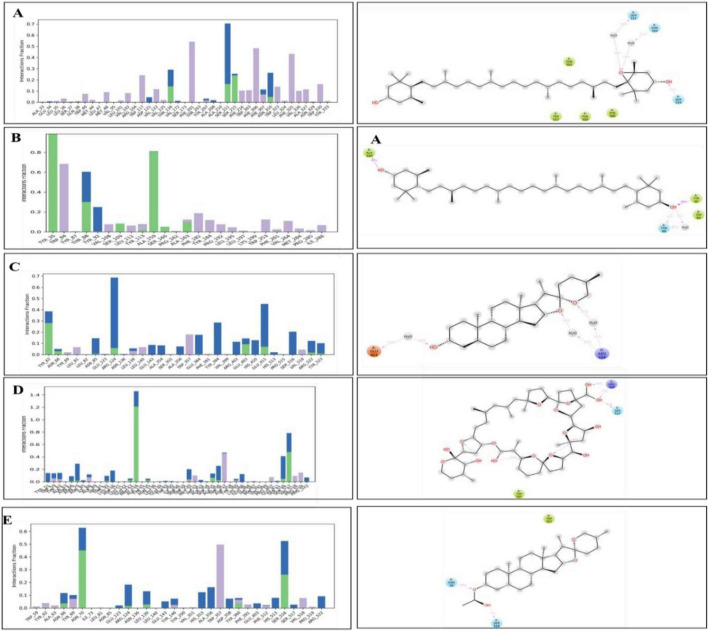
Hydrogen bond occupancy profiles of cardiovascular disease (CVD) protein–ligand complexes. The left-side bar graphs represent the normalized frequency of hydrogen bonds (green), water bridges (blue), and hydrophobic interactions (purple) formed between the protein backbone and the ligand atoms during molecular dynamics simulations. The right-side panels display the corresponding 2D interaction diagrams showing the spatial orientation of ligand binding with the critical binding residues **(A)** β1AR–antheraxanthin, **(B)** hATRs–zeaxanthin, **(C)** ACE–diosgenin, **(D)** ACE–pectin, and **(E)** ACE–yamogenin acetate.

**FIGURE 5:**
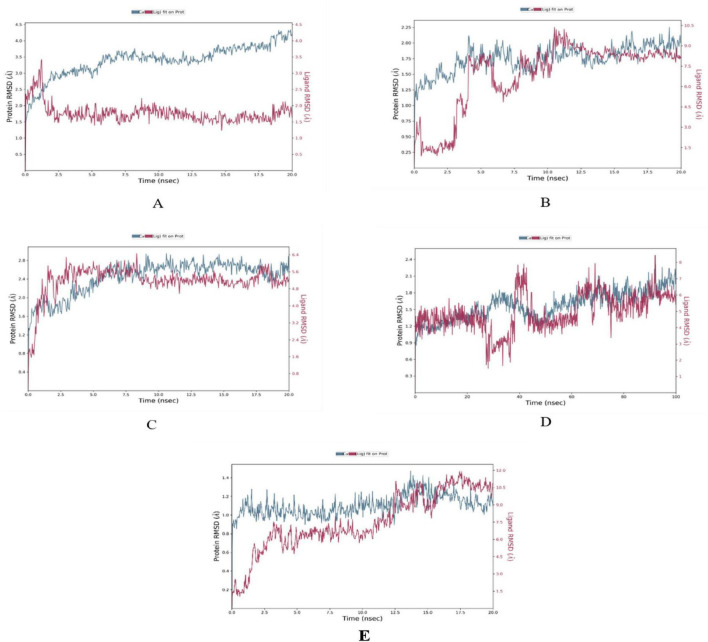
Root Mean Square Deviation (RMSD) plots of T2DM-related protein–ligand complexes generated from molecular dynamics simulations. The RMSD profiles display the structural stability of the protein backbone (blue line) and the ligand (red line) over time. **(A)** SGLT1–antheraxanthin, **(B)** DPP-IV–diosgenin, **(C)** DPP-IV–pectin, **(D)** AR–yamogenin acetate (100 ns), and **(E)** AR–zeaxanthin. The Y-axis represents RMSD values in Å (Ångström), indicating the extent of deviation from the reference structure. Consistently low or stabilized RMSD values suggest favorable and stable ligand binding to each T2DM target.

The interaction analysis corroborated several hydrogen bond interactions observed in earlier studies. The binding of antheraxanthin to β1AR and zeaxanthin to hATRs, with key residues involved in hydrogen bonding (e.g., Ser215, Ser126, Ser211), was consistent with previously identified interactions in related receptors (44) (Figure 4). However, the interaction of ACE with diosgenin, which showed hydrogen bonding with Tyr62 and Glu411 residues, is in line with studies that also noted ACE’s flexibility and ability to form multiple binding sites for different ligands (45) (Figure 4C). The average RMSD of SGLT1 was 3.5 Å, indicating backbone stability, while the RMSD of antheraxanthin (2.0 Å) suggests stable binding to SGLT1 (Figure 5A).

Furthermore, the stability observed in human DPP-IV with diosgenin, which had RMSD values averaging 7.5 Å, supports findings from simulations on DPP-IV inhibitors (46), demonstrating that higher RMSD values can reflect varying degrees of ligand affinity and complex stability across different simulation setups (Figure 5B). RMSD analysis of human DPP-IV revealed a stable protein–pectin complex, with the protein maintaining an RMSD of approximately 2.5 Å and pectin averaging 5.0 Å (Figure 5C). The lack of significant fluctuations in the human AR complex with yamogenin-acetate and zeaxanthin (1.5–7.5 Å) further aligns with previous research highlighting the role of these ligands in stabilizing the protein structure (47) (Figures 5D,E).

**FIGURE 6:**
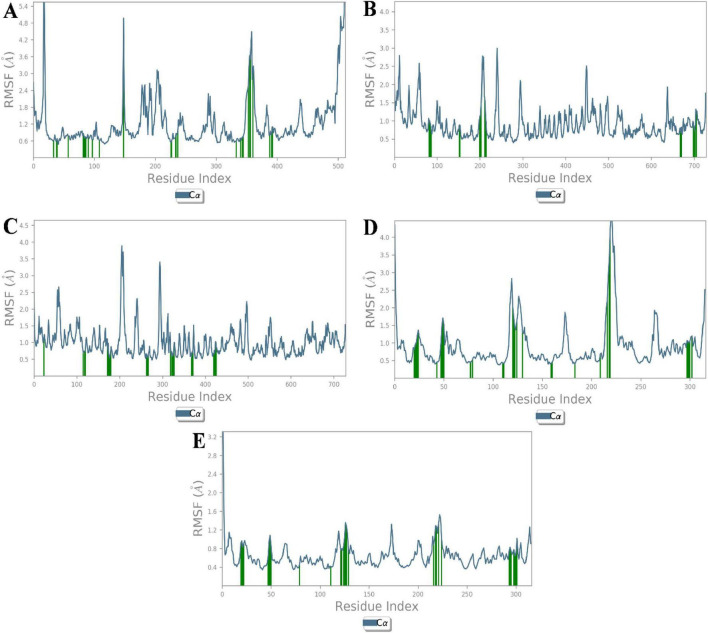
Root Mean Square Fluctuation (RMSF) analysis of type 2 diabetes mellitus (T2DM)-related protein–ligand complexes. The RMSF values represent the average positional deviations of Cα atoms of each residue over the simulation time, providing insight into the flexibility of different protein regions. Higher peaks in the graph represent the flexible regions of protein structure. **(A)** SGLT1–antheraxanthin, **(B)** DPP-IV–diosgenin, **(C)** DPP-IV–pectin, **(D)** AR–yamogenin acetate, and **(E)** AR–zeaxanthin. During molecular dynamics simulations, reduced fluctuations within the active site regions suggest stable binding interactions between the phytochemicals and their respective T2DM targets.

The RMSF analysis of T2DM-docked complexes revealed specific fluctuations in the proteins. For SGLT1-antheraxanthin (Figure 6A), deviations were observed in the N-terminal and central regions (residues 15–22, 148–152, 178–188, 349–368, 465–511). The DPP-IV-diosgenin complex (Figure 6B) showed minor fluctuations around residues 62–73, 201–215, 233–244, 291–305, and 455–465. The DPP-IV-pectin complex (Figure 6C) showed minimal fluctuations, indicating stability. In the AR-yamogenin acetate complex (Figure 6D), deviations were observed around residues 120–130 and 221–232, while the AR-zeaxanthin complex (Figure 6E) remained stable, with no significant deviations during the simulation.

**FIGURE 7:**
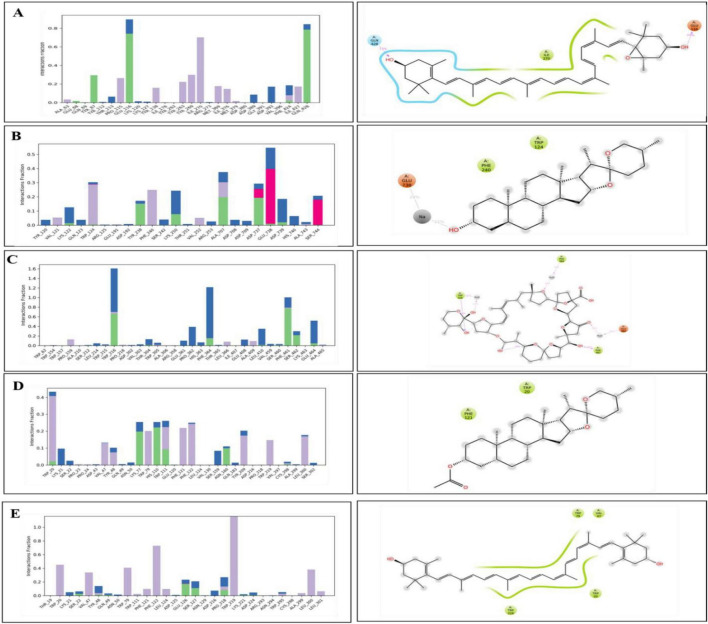
Hydrogen bond occupancy profiles of type 2 diabetes mellitus (T2DM)-related protein–ligand complexes. The bar plots (left) depict the relative frequency of hydrogen bonds (green), water bridges (blue), and hydrophobic interactions (purple) between the ligands and protein backbone residues throughout the molecular dynamics simulations. The accompanying 2D interaction diagrams (right) illustrate the spatial orientation of ligand binding and key contact residues within the active sites of each target. **(A)** SGLT1–antheraxanthin, **(B)** DPP-IV–diosgenin, **(C)** DPP-IV–pectin, **(D)** AR–yamogenin acetate, and **(E)** AR–zeaxanthin.

Hydrogen bond analysis confirmed key interactions for complex stability. SGLT1-antheraxanthin (Figure 7A) formed hydrogen bonds with Glu-116 and Gln-428 and hydrophobic interactions at Ile-270. DPP-IV-diosgenin (Figure 7B) showed ionic bonds at Glu-738 and hydrophobic interactions at Trp-124 and Phe-240. DPP-IV-pectin (Figure 7C) had hydrogen bonds at Trp-216 and Phe-461. AR-yamogenin acetate (Figure 7D) and AR-zeaxanthin (Figure 7E) showed hydrophobic interactions at key residues, confirming stability. These results align with previous studies (48, 49). Variations in RMSD and RMSF values suggest unique phytochemical interactions, offering the potential for tailored CVD and T2DM therapies.

### 3.5 Molecular mechanics generalized born surface area (MM/GBSA) calculations

The molecular mechanics with generalized Born and surface area solvation (MM/GBSA) technique was employed to calculate the average binding free energy (ΔG) of the top protein-ligand complexes at 100 ns (Table 6). The ligand zeaxanthin showed a decrease in energy of −67.29 kcal/mol and −64.22 kcal/mol (100 ns) when bound to the human AR receptor and hATRs protein, respectively. The compounds zeaxanthin, antheraxanthin, yamogenin acetate, and diosgenin had the lowest values for VDW and hydrogen bonding energies, indicating that these compounds possess the most potential for binding with active site residues.

**TABLE 6:** Molecular mechanics generalized born surface area (MM/GBSA) binding free energy of top complexes (kcal/mol).

Name of complex	vdW	Coulomb	Covalent	Hbond	ΔGTotal (kcal/mol)
3F8S-Pectin	−50.22	−4.54	6.88	−0.86	−48.74
108A-Pectin	−47.64	−3.22	2.14	−0.53	−49.25
108A-yamogeniacetat	−53.57	0	2.45	−0.32	−51.44
108A-diosgenin	−48.75	0	1.24	−1.14	−48.65
2YCW-antheraxanthin	−64.45	−3.31	18.45	−0.35	−49.66
3DH4-antheraxanthin	−65.33	−6.35	11.15	−1	−61.53
3S3G-zeaxanthin	−66.96	−10.08	10.12	−0.37	−67.29
3S3G-yamogenin acetate	−38.45	−1.66	4.26	−0.4	−36.25
3F8S-diosgenin	−32.8	−11.9	1	−0.5	−44.2
4YAY-zeaxanthin	−65.6	0	1.76	−0.38	−64.22

### 3.6 Physicochemical and toxicological properties of selected phytochemicals

**FIGURE 8:**
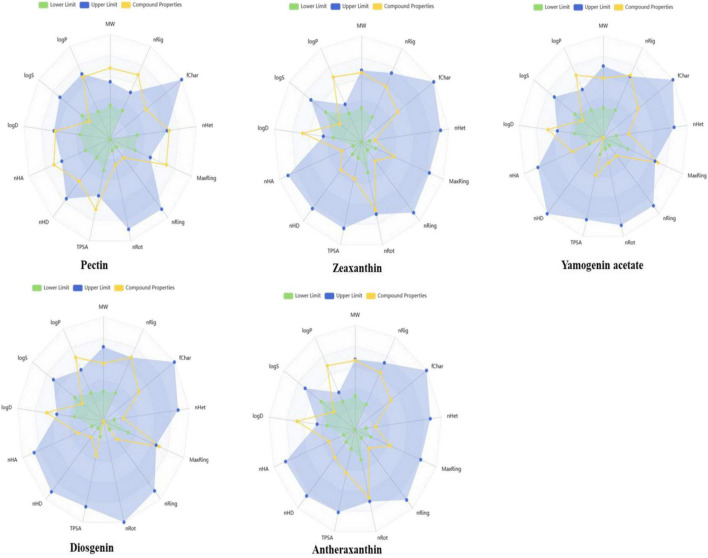
Radar plots illustrating the predicted physicochemical properties of selected date vinegar phytochemicals: pectin, zeaxanthin, yamogenin acetate, diosgenin, and antheraxanthin. Each plot compares the compound’s calculated properties (yellow lines) with established drug-likeness thresholds—green representing lower acceptable limits and blue representing upper limits. Properties include molecular weight, hydrogen bond donors and acceptors, topological polar surface area (TPSA), lipophilicity (logP), and solubility parameters. All compounds fall within or near the optimal drug-likeness range, supporting their suitability for bioactivity and absorption.

The selected phytochemicals were also analyzed to calculate the physicochemical properties, Lipinski rule of five, and toxicity analyses. The properties include molecular weight (MW g/mol), hydrogen bond acceptor (nHA), hydrogen bond donor (nHD), polar surface area (TPSA), logP, rotatable bonds (nRot), rings (nRing), heteroatoms (nHet), atoms in the biggest ring (MaxRing), the logarithm of aqueous solubility (LogS), and the logarithm of n-octanol (logD) were determined. The compound properties with lower and upper limits are presented in the radar view images (Figure 8). It was further observed that the cyclic compounds showed significant biological and drug properties.

### 3.7 Sugar dynamics and bioactive retention in dates under drying conditions

Different PVD temperatures were applied to assess the sugar dynamics (Table 7). The total sugar concentration of the fresh sample was 33.59%, which served as a baseline for comparison. Drying at 65°C resulted in a sugar concentration of 53.68%; at 70°C, the concentration reached 55.13%. However, drying at 75°C caused a significant drop in sugar concentration to 34.43%, close to the fresh sample’s value. Although higher drying temperatures, such as 75°C, reduce sugar content, they may also lead to thermal degradation of bioactive compounds. This suggests that while 75°C is beneficial for reducing sugar, it may compromise the preservation of bioactive properties, consistent with findings showing that higher temperatures can degrade bioactive compounds while reducing sugars in fruit drying (7). In contrast, moderate drying temperatures (65 and 70°C) may increase sugar concentration but preserve more bioactive compounds.

**TABLE 7:** Total sugar content of red dates: ANOVA and Duncan’s test results.

Sample condition	Sugar (%)	*p*-value (ANOVA)	Group (Duncan’s Test)
PVD-65°C15:4	53.68	<0.0001	A
PVD-70°C15:4	55.13	<0.0001	A
PVD-75°C15:4	34.43	<0.0001	B
Fresh sample	33.59	<0.0001	B

Sugar content (%) of red dates showed statistically significant differences based on ANOVA (*p* < 0.0001). According to Duncan’s multiple range test, the samples were grouped into: Group A (PVD-65°C 15:4, PVD-70°C 15:4) and Group B (PVD-75°C 15:4, fresh sample).

ANOVA showed a significant difference in total sugar content across the four sample conditions (*p* < 0.0001), indicating that drying temperature affects sugar retention. Duncan’s test revealed that PVD-65C°15:4 and PVD-70C°15:4 had similar sugar contents (Group A), while PVD-75C°15:4 and fresh samples had significantly lower sugar content (Group B). This suggests that higher drying temperatures lead to reduced sugar retention.

### 3.8 Mechanisms and therapeutic potential of red date vinegar in CVD and T2DM

This study demonstrates the therapeutic potential of red date vinegar in managing glucose homeostasis, lipid metabolism, and cardiovascular health through multiple integrated mechanisms. By activating AMPK, inhibiting SGLT1, downregulating HMG-CoA reductase, and upregulating ApoA-I, red date vinegar supports improved glycemic control and lipid profiles, targeting key pathways involved in T2DM and CVD.

Fresh dates, due to their balanced sugar profile and high content of bioactive compounds, may serve as an optimal substrate for vinegar production. Although individuals with diabetes often avoid dates due to their high natural sugar content, converting dates into vinegar through fermentation offers a promising alternative that may alleviate glycemic concerns while preserving beneficial phytochemicals.

Significantly, the fermentation process enhances the bioactivity of date vinegar and reduces its glycemic impact, making it a potentially safer option for diabetic populations. For example, total sugar content can be significantly reduced during fermentation, with one study reporting a decrease from 231.17 to 15.89 g/L over 12 days, alongside a rise in alcohol content from 0.76 to 9.75% v/v due to yeast metabolism (50). This process may also enhance the bioavailability of health-promoting compounds such as polyphenols and antioxidants, reinforcing the potential of fermented date vinegar as a functional food for metabolic regulation. However, these findings warrant further validation under standardized fermentation protocols and across diverse clinical settings to ensure safety and efficacy in diabetic management.

**FIGURE 9:**
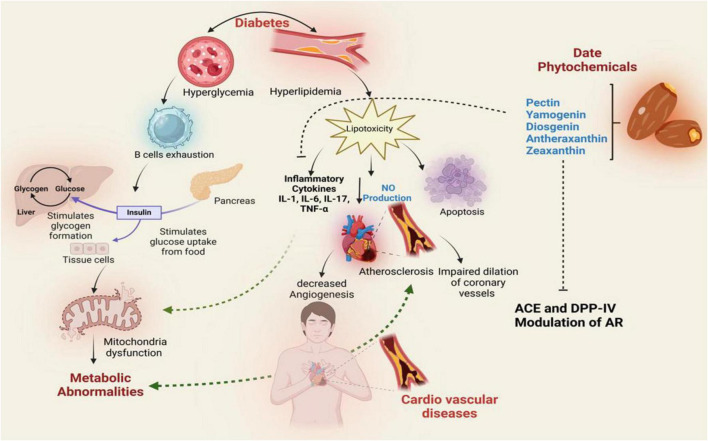
Proposed mechanistic pathway illustrating the therapeutic effects of date vinegar phytochemicals (pectin, yamogenin, diosgenin, antheraxanthin, and zeaxanthin) on diabetic and cardiovascular complications. The diagram integrates their potential actions on insulin signaling, glucose metabolism, lipid regulation, and inflammation. Phytochemicals modulate key targets such as ACE, DPP-IV, and AR, reducing hyperglycemia, hyperlipidemia, and cytokine-mediated inflammation. These interactions promote nitric oxide (NO) production, inhibit lipotoxicity and apoptosis, and improve mitochondrial function, thereby mitigating endothelial dysfunction, atherosclerosis, and cardiovascular risk.

Molecular docking and dynamics simulations further revealed stable interactions between red date vinegar-derived compounds—pectin, yamogenin acetate, diosgenin, antheraxanthin, and zeaxanthin—and key targets including ACE, SGLT1, DPP-IV, β1AR, and AR. These interactions support inhibition of ACE and DPP-IV, modulation of AR, reduced oxidative stress via Nrf2/HO-1 activation, and anti-inflammatory effects through suppression of IL-6 and TNF-α (Figure 9).

In addition, the favorable drug-likeness properties of these compounds, as determined by Lipinski’s rule of five, and the alignment between *in silico* predictions and experimental data, underscore their potential as dual-target therapeutic agents in the management of both T2DM and CVD.

## 4 Conclusion

This study supports the potential of red date vinegar as a functional dietary intervention for managing type 2 diabetes mellitus and cardiovascular disease. The research highlights its therapeutic relevance by integrating computational predictions with experimental findings. The modulation of key metabolic and inflammatory pathways demonstrates its ability to target the complex pathophysiology of these chronic disorders, thereby offering a foundation for future *in vivo* studies and clinical applications.

## Data Availability

The original contributions presented in this study are included in this article/supplementary material, further inquiries can be directed to the corresponding authors.
